# Case Report: A five-year follow up after pediatric renal transplantation using flow cytometry crossmatch and HLA immunophenotyping based on DNA for screening test

**DOI:** 10.12688/f1000research.51407.1

**Published:** 2021-05-06

**Authors:** Johanes Dwi Meiyanto, Besut Daryanto, Kurnia Penta Seputra

**Affiliations:** 1 Urology, Faculty of Medicine, Universitas Brawijaya – Saiful Anwar General Hospital, Malang, East Java, 65145, Indonesia

**Keywords:** flow cytometry crossmatch, HLA based on DNA, pediatric renal transplantation

## Abstract

**Background: **There are three methods for renal replacement therapy for end stage chronic kidney disease; dialysis, continuous ambulatory peritoneal dialysis, and renal transplantation which is the best because of the least morbidity rate, the best survival rates, the best quality of life, and the best improvement in activities of daily living. In the field, flow cytometry serves a well-established role in pre- and post-transplant crossmatching, and if it is combined with human leukocyte antigen (HLA) immunophenotyping based on DNA, it will produce a more sensitive prediction of the chronic graft rejection compared to complement-dependent cytotoxicity crossmatching and can eliminate irrelevant antibody (IgM). This is the first experience using this method in our hospital. The survival rate at one, five and ten years has been shown to be 99%, 97% and 96%, respectively; therefore, we wanted to find out the five year follow up of the patient.

**Case presentation: **We evaluated a 20-year-old female with a history of pediatric renal transplantation five years previously due to end stage renal disease caused by bilateral parenchymatous renal disease. She had a history of hypertension since December 2014 and underwent hemodialysis for three months. The transplantation took place in March 2015. A kidney from her mother was transplanted to recipient using end-to-side anastomoses. After five years, the patient was routinely monitored at the urology clinic, with creatinine serum results between 1.5 and 2 mg/dL, urea and electrolyte serum levels within normal limits and she could resume normal life.

**Conclusions: **Survival five years after the procedure showed a beneficial outcome of the method used.

## Background

End stage renal disease (ESRD) is a relatively rare case in children with limited epidemiological data. In 2008, the prevalence of this disease was approximately 65 per million age-related populations in Australia, Canada, Malaysia and Western Europe. ^1^^,^^2^ Dialysis, continuous ambulatory peritoneal dialysis (CAPD), and renal transplantation are three options for renal replacement therapy in ESRD. Renal transplantation is the preferred method of treatment for ESRD patients despite the risk of body rejection reactions as complications of the surgery, because of the least morbidity rate, the best survival rates, the best quality of life, and the best improvement in activities of daily living. ^3^^–^^5^

Flow cytometry crossmatch is a standard technique for evaluating the recipient’s and donor’s compatibility for renal transplants. The recipient’s serum, together with donor lymphocytes, is incubated and analyzed in a flow cytometer for the presence of IgG antibodies. ^6^ Flow cytometry plays an important role in crossmatch before and after transplantation. When combined with human leukocyte antigen (HLA) immunophenotyping based on DNA, flow cytometry will have a more sensitive prediction for chronic graft rejection than complement-dependent cytotoxicity (CDC) crossmatching and is able to eliminate irrelevant antibodies (IgM). ^7^

Flow cytometry crossmatch can inform several things such as cytokine production in intracellular, analysis of phenotyping, and detailed information related to several T cells subset. By using tagged multimers with fluorescent, we could specifically calculate cellular antigen stimulation test (CAST). We can find MHC Class I or Class II monomers in multimers. ^8^

The American Family Children's Hospital showed that the survival rates for post renal transplantation pediatric patients after 11 months, 1 year, and 3 years were 100%, 98%, and 97%, respectively. Meanwhile, graft survival is very dependent on the success of the renal transplantation. When compared with other therapeutic modalities, the prevalence of renal transplantation survival at 1 year was quite high at 97.9%, compared with CAPD (94.5%) and hemodialysis (87.3%). Over a longer period of time, the survival rate for post renal transplantation patients at 10 years was 86%, compared with CAPD (35%) and hemodialysis (33.8%). ^9^

In our first experience of renal transplantation using flow cytometry screening and HLA immunophenotyping, we wanted to know the results at five years follow-up after transplantation.

## Case presentation

We present the case of a 20-year-old Javanese woman who underwent renal transplantation five years previously due to ESRD caused by bilateral parenchymatous renal disease. She is a student.

**Figure 1.**
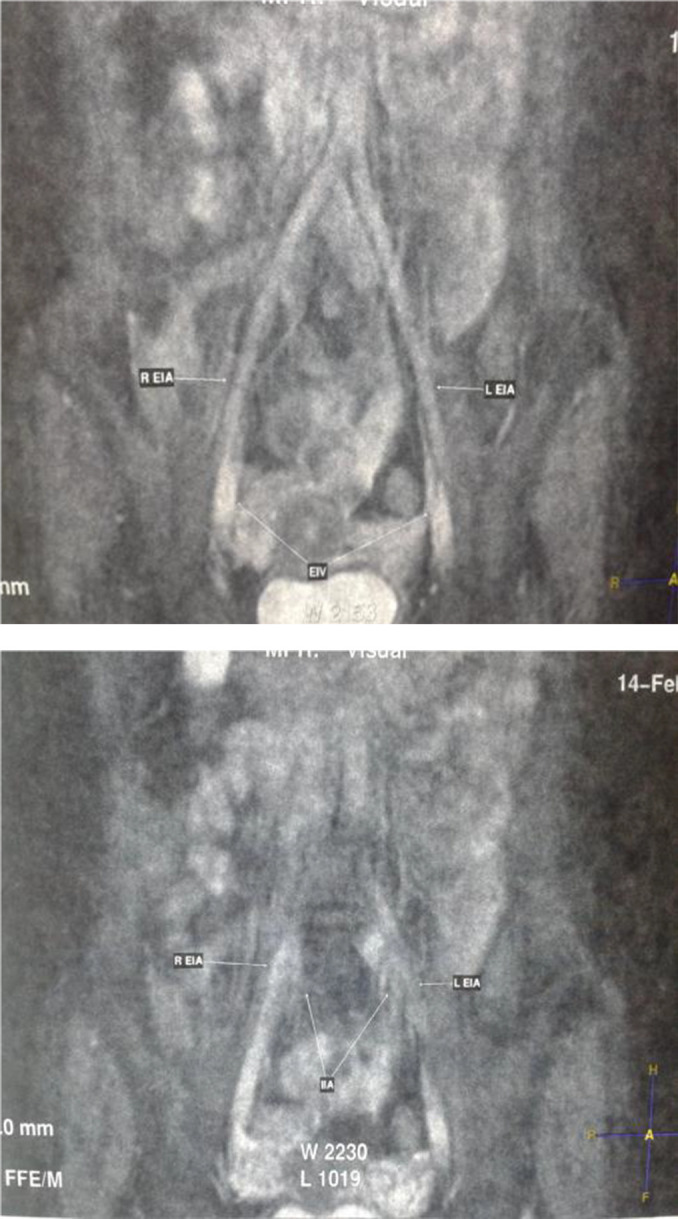
Patient’s preoperative magnetic resonance angiography.

She had a history of hypertension since December 2014 and had undergone hemodialysis for three months. She came to the hospital with right and left lower back pain for the past year. She had high blood pressure of 140/90 mmHg, anemic conjunctivas and bilateral flank pain. Initial laboratory tests showed hemoglobin levels of 7.3 g/ dL, leukocytes 4750/ mm ^3^, platelets of 174,000/mm ^3^, blood urea nitrogen (BUN) 16.3 mg/ dL and serum creatinine 4.21 mg/dL. Anti-CMV IgG showed reactive results and a complete urine examination found albuminuria (2+). On radiological examination, chest radiograph and plain abdominal radiograph were within normal limits. Chest radiograph showed that heart in normal shape, size and position; lung in normal vascularization, bilateral hilum in good condition, and there were no infiltrate, cavity or nodul; there was no abnormality of aorta; trachea in normal position; costophrenic angle was sharply-pointed; bilateral hemidiaphragm in normal dome shaped; bones and soft tissues were normal. Abdominal radiograph showed that bilateral pre-peritoneal fat lines and psoas lines in normal condition; normal intestinal gas pattern; bones and soft tissues were normal. Abdominal ultrasound showed an increased echogenicity of bilateral renal cortex that could reflect a bilateral parenchymatous renal disease. Magnetic resonance angiography of the recipient showed bilateral decreased parenchymal thickness that reflected chronic parenchymatous renal diseases; ascites in the pelvic cavity; left and right common iliac arteries, internal iliac artery, external iliac artery were in normal vascularization and showed no thrombus or stenosis ( Figure 1).

**Table 1.** HLA-ABDR typing (donor-recipient) examination results with PCR-SSP method. PCR-SSP: polymerase chain reaction-based sequence specific primers; HLA: human leukocyte antigen; ABDR: AB serology and oligotyping for DR114.

	HLA - A		HLA-B		HLA-DR	
	*Serotype*	*High resolution*	*Serotype*	*High* *resolution*	*Serotype*	*High* *resolution*
**Donor**	A11, A33	A* 11:01//A*33:01	B44, B54	B* 44:02:07//B*54:14	DR04, DR07	DRB1* 04:01//DRB1*07:01
**Recipient**	A02, A33	A* 02:01//A*33:01	B44, B15	B* 44:02:07//B*15:01	DR07, DR15	DRB* 07:01//DRB1*15:01

**Figure 2.**
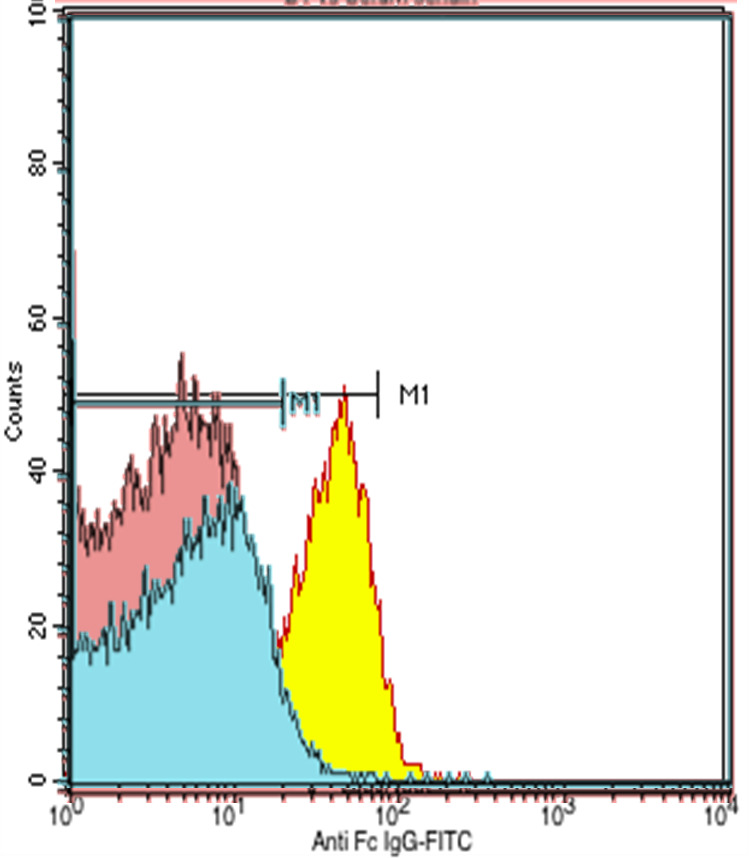
The negative results of the T-lymphocyte flow cytometry examination.

The patient was subsequently diagnosed with stage V chronic kidney disease based on history of hypertension, high blood pressure (140/90 mmHg), anemic condition (HB: 7.3gr/dL) and elevated renal function test (urea 16.3 mg/dL and serum creatinine 4.21 mg/dL) and decreased glomerular filtration rate (GFR: 12,97 ml/menit/1,73m ^2^). Differential diagnosis were diabetic nephropathy, chronic glomerulonephritis, nephrosclerosis, polycystic kidney disease, rapidly progressive glomerulonephritis but all other diagnosis had been excluded from the radiologic examination and no previous history of diabetes mellitus and no sign of hyperglycemia. She had undergone hemodialysis for three months. Flow cytometry and DNA-based HLA testing between the recipient and the donor was carried out to assess prediction of graft rejection prior to renal transplantation surgery. The HLA screening showed mismatch typing 3/6 and flow cytometry of T and B lymphocytes was negative.

The renal transplantation was performed in March 2015 by urologists from Malang (Malang’s Kidney Transplantation Team). Before the procedure, the patient had been optimized by hemodialysis prior surgery. Patient was under general anasthesia and in supine position. The transplantation procedure used the end-to-side anastomoses method on the donor renal artery with the right external iliac vein. The ureter from the donor kidney was neo-implanted into the recipient bladder using the Lich Gregoir technique, and a special double-J (DJ) stent No. 12 and retroperitoneal drain was placed. Doppler ultrasound examination was performed to assess the vascularization of the new kidney in the recipient's body. The result showed that the color flow didn’t reach the periphery, vascularization of the interlobular arteries was good and the surgical wound was closed. It turned out that there was tension in the fascia, reflected in abnormal resistive index and disproportion of larger size of the donor kidney to the smaller space in the recipient’s pelvic cavity and this resulted in the decreased blood flow to the kidneys seen in Doppler ultrasound evaluation. Then we decided to open the stitches and after that, ultrasound evaluation was carried out, which showed an improvement in blood flow. Finally, a mesh was placed on the recipient's fascia with the aim of reducing tension, then the wound was closed and reexamined with Doppler ultrasound, which showed that the color flow reached the periphery and the interlobular artery could be visualized without Doppler power ( Figure 4).

**Figure 3.**
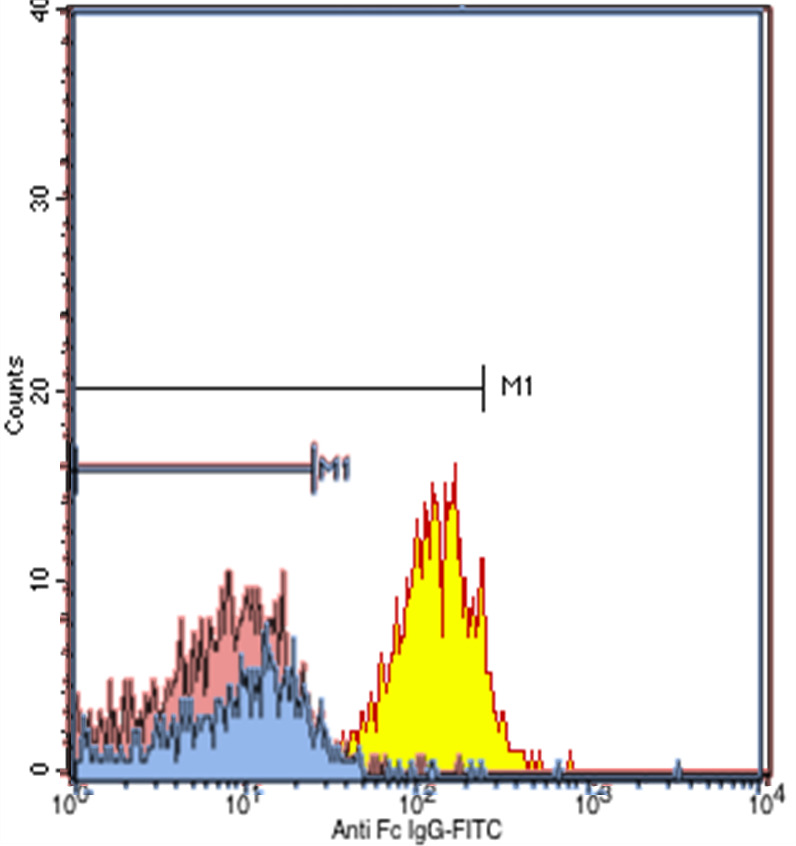
The negative results of the B-lymphocyte flow cytometry examination.

**Figure 4.**
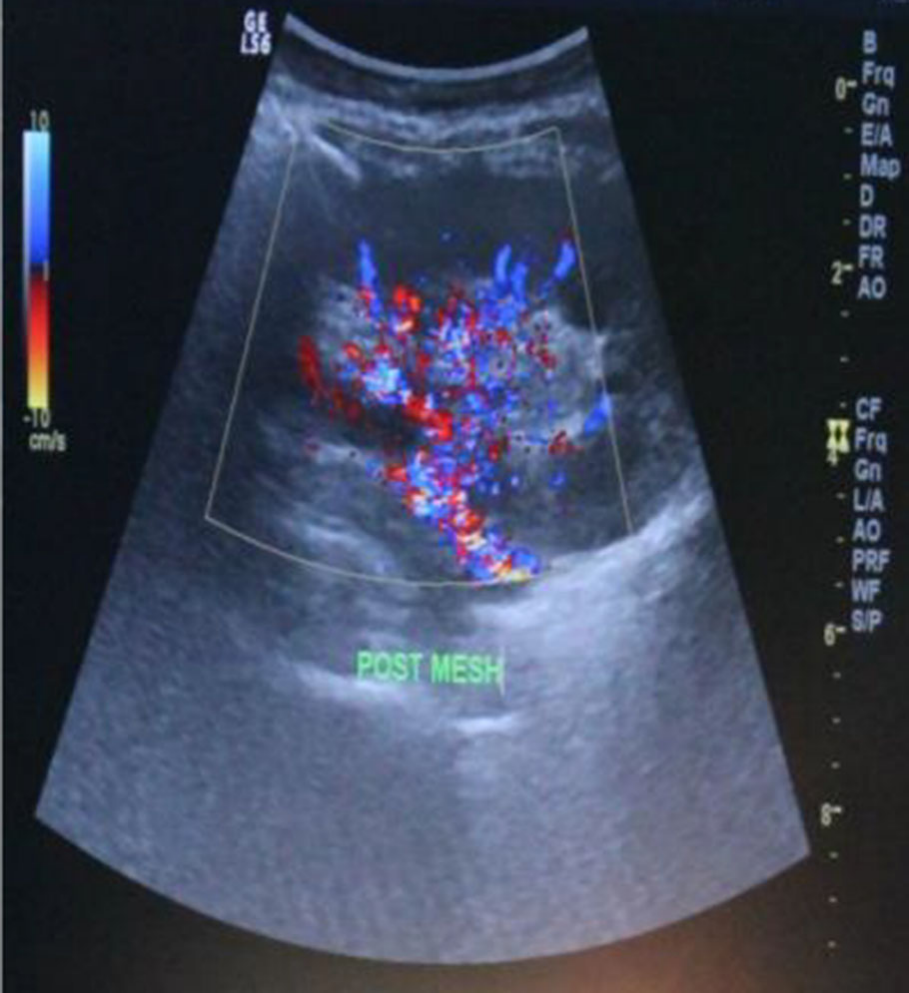
Ultrasound evaluation results of recipient after mesh insertion.

**Figure 5.**
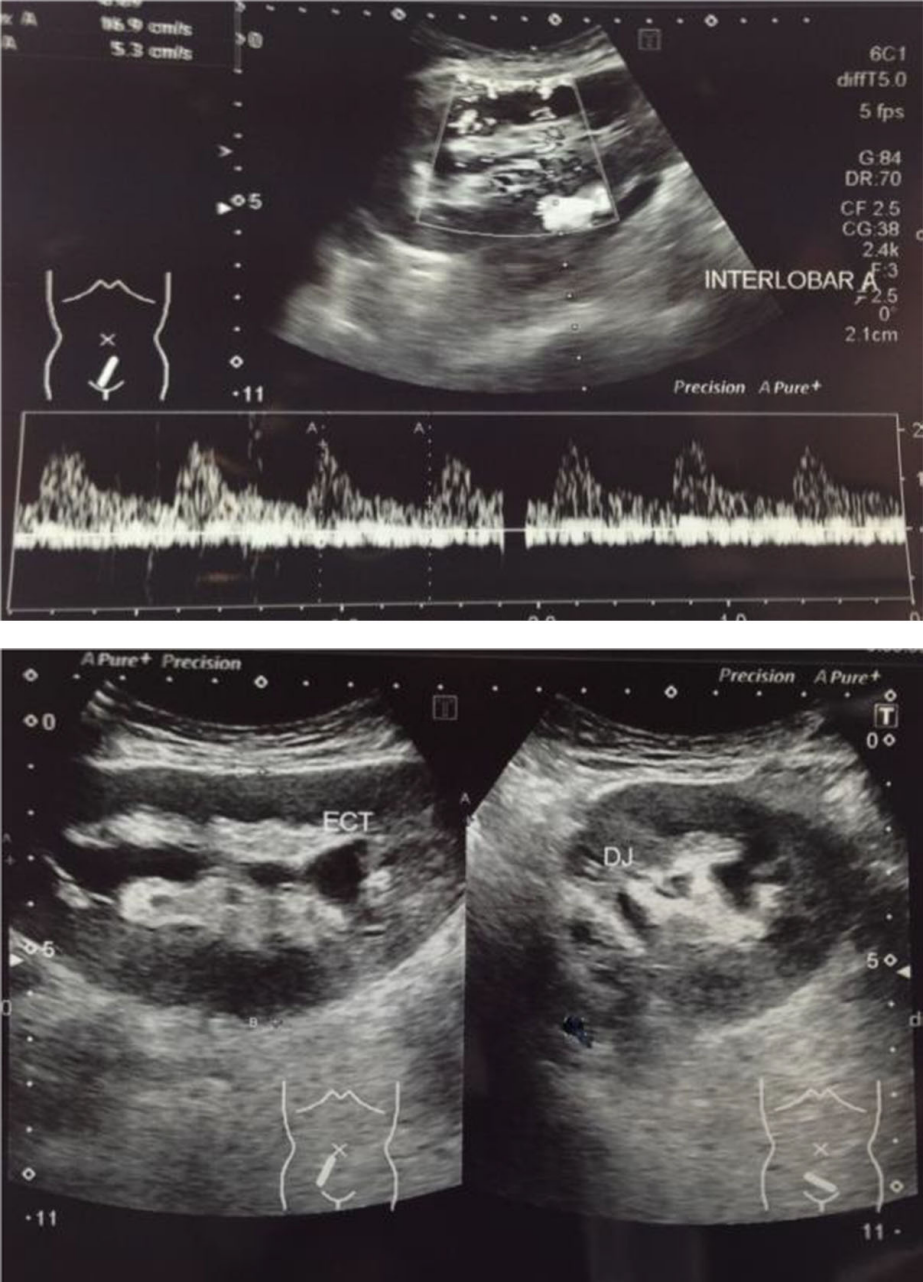
Ultrasound evaluation results of recipient after double-J stent insertion.

**Figure 6.**
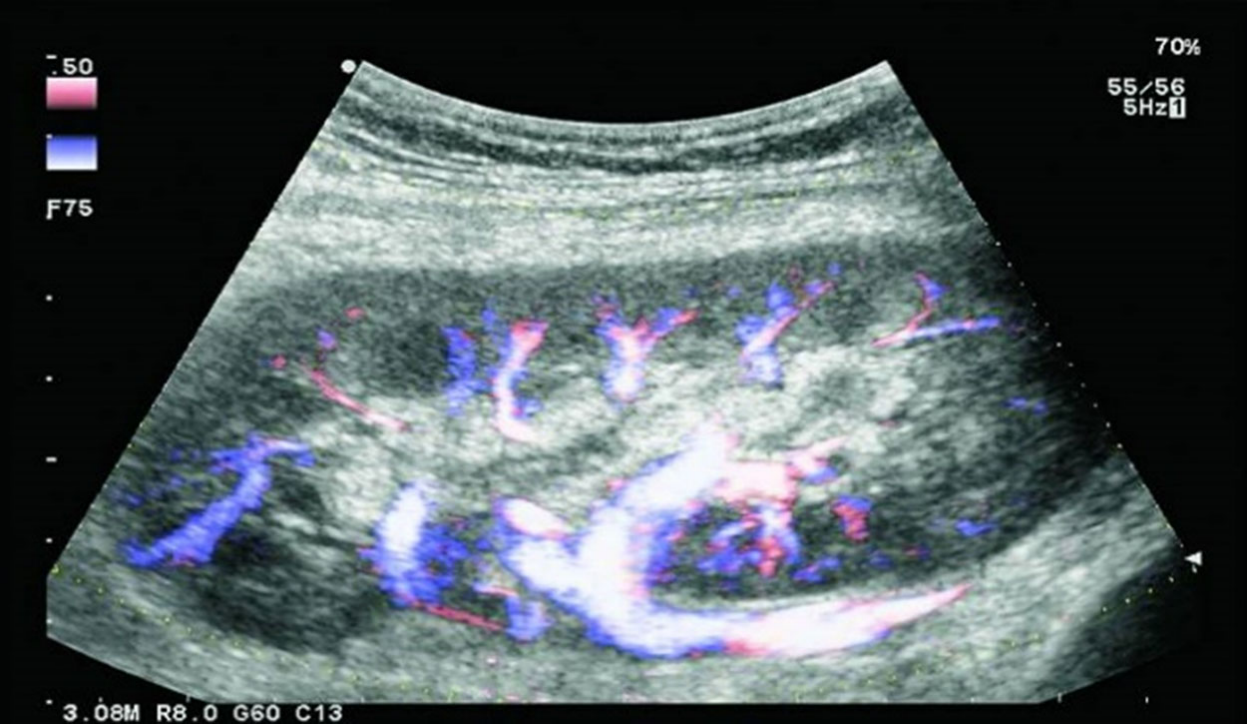
Renal ultrasound five years after transplantation.

After the surgery, the recipient was directly admitted to the intensive care unit for five days with strict observation of fluid balance, electrolyte imbalance, blood pressure, and related laboratory parameters. After transplantation, the patient was given medication in the form of 500 mg Cellcept two times a day and a 5 mg Prograft two times a day as immunosuppressants. After two weeks of hospitalization, the patient showed good response to immunosuppressant drugs and showed no rejection reaction to transplantation. Then she was allowed to discharge from hospital at the early of April 2015 after two weeks in ward. She was asked to come back to urology clinic in the same hospital one month after discharged.

A month later the patient came to urology clinic for evaluation of DJ-stent removal and it was done under local anasthesia by urologist from Malang. She was asked to come back at three months. Then she came again at three months with good results. When the patient returned for monitoring at the urology clinic at four months, the creatinine level had increased by 4.9 g/dl and the ultrasound results showed hydronephrosis grade II. It was then decided to redo surgery to find out the cause of hydronephrosis. The patient undergone hemodialysis prior surgery to optimize her condition. The surgery was done under general anesthesia and in lithotomy position. The patient underwent a ureterorenoscopic evaluation and right ureteral stenosis was found during the procedure. Then she underwent a ureterorenoscopy with a Holmium laser to dilate the ureteral orifice and a DJ stent was re-inserted into the patient. She was hospitalized for two days and discharged in the early of July 2015 after being two days in ward. She was asked to come back to urology clinic one month after discharged.

A month later the patient came to urology clinic for the evaluation of DJ-stent removal. She was in a good condition and the ultrasound results showed that the size of transplanted renal was 10.1 cm × 5.6 cm with normal echocortex, hydronephrosis grade I, DJ-stent insitu, cortex-medulla border in normal condition, no sign of ureteral widening, and doppler parameters in normal condition ( Figure 5).

She could do her usual activities of daily living three months after surgery, and during these three months she only did light activities in her house. After that she came routinely to urology clinic every month to monitor and evaluate her condition, laboratory and other supporting examination results.

After five years post transplantation, the patient returned to outpatient care for routine appointments without any complaints. She also routinely consumed Herberser 100 mg once a day, Cellcept 500 mg twice daily, Prograf 0.5 mg once a day, Methyl Prednisolone 4 mg every two days, and Simvastatin 20 mg once a day. There were no abnormalities on physical examination. The latest laboratory test results showed a haemoglobin level of 11.9 g/dL, erythrocytes 4.790/uL, leukocytes 7,000/uL, hematocrit 38%, platelets 325,000/uL, iron 34 ug/dL, total iron binding capacity 301μg/dL, transferrin saturation 11%, albumin 4.3 g/dL, fasting blood glucose 93mg/dL, urea 33 mg/dL, creatinine 1.44 mg/dL, uric acid 5.9 mg/dL, sodium 140mmol/L, potassium 3.9 mmol/L, chloride 106 mmol/L, phosporous 3.3 mg/dL, and tacrolimus 3.8 ng/dL (tacrolimus levels in the last five years ranged from 3,5-7 ng/dl). Ultrasound examination of the patient revealed intrarenal vascularization, transplanted arteries and veins, and iliac arteries within normal limits ( Figure 6). In these five years, the patient was routinely monitored at the urology clinic with serum creatinine levels between 1.5 to 2 mg/dL, routine hematology, albumin levels, serum urea, electrolytes, and tacrolimus levels within normal limits. She was able to carry out normal daily activities and is currently continuing her education at university.

## Discussion

Renal transplantation is the preferred treatment option in children with ESRD. Renal transplantation is currently considered the treatment option of ESRD for children because it is associated with better quality of life, productivity, and child growth, as well as longer survival than other modalities. There have been significant developments related to renal transplantation, including immunosuppressive medication therapy, grafting, and patient safety. These developments can contribute to many factors, including pre-transplant care, surgical development, and stronger immunosuppressant management. ^10^^,^^11^

HLA protein antigen on the cell surface is the key to differentiation of self-proteins from non-self-proteins. The two groups of MHC genes relevant to transplantation are class I and class II. HLA matching is a known predictor for the success of renal allograft. HLA typing is a crucial step in renal transplantation because recognition of foreign HLA by recipient T lymphocytes will trigger an immune response. Activation of T lymphocytes initiates a cascade of mediators that causes the immune system to fight allografts. ^12^^,^^13^

Flow cytometry crossmatch, as well as CDC crossmatching, also depends on donor lymphocytes which incubated with the receiving serum. Nevertheless, instead of a complement factor addition, a fluorescence coated second antibody was added which acted against IgG-DSA. These anti-IgG antibodies bind to the complex of donor Ag-DSA and allow the flow cytometer to detect. The result of reading is semi-quantitative and more objective because these antibodies instead of quantifying cell death visually, use a channel shift over the baseline. Flow cytometry crossmatch is more sensitive than CDC crossmatching because it does not depend on activity of complement. Negative value of flow cytometry crossmatch excludes the possibility of immunologically significant DSA. Furthermore, flow cytometry crossmatch is also more specific than CDC crossmatch because it only detects IgG antibodies and does not detect IgM. ^14^

A study has shown that flow cytometry has become the most sensitive approach for detecting HLA alloantibodies. ^15^ The anti-human globulin-supplement dependent cytotoxicity (AHG-CDC) method provides a higher antibody detection sensitivity than CDC method. However, this technique is limited because of its low lymphocyte viability. On the other hand, flow cytometry crossmatch does not require cell viability or depend on complement fixation, but can detect immunoglobulin molecules that bind to target cells using immunofluorescence. Thus, low lymphocyte viability did not affect the results of flow cytometry crossmatch. In addition, flow cytometry crossmatch can detect not only complement-binding antibodies but also immunoglobulins that correct incompleteness. Therefore, flow cytometry crossmatch is more sensitive than CDC and AHG-CDC. Previous reports on a population in Thailand found that flow cytometry crossmatch was 8-32 times and 4-16 times more sensitive than CDC and AHG-CDC. They also found 28.9% of flow cytometry crossmatch to be positive in patients with negative CDC. ^16^

The strengths in this case were:

1. This case was the first five year follow up of pediatric renal transplantation using flow cytometry crossmatch and HLA immunophenotyping based on DNA for screening test in our hospital. So that we could know more how this procedure bring good hope for the patient and the donor, also how was the survival rate after five years, whether it followed the theory or not.
2. A pilot project for other patient with the same procedure which had underwent procedure after this case to be followed and monitored for the survival rate.

The limitations in this case were:

1. Only one patient had been followed up in this case report, leading to lack of data and needed more considerations and more patients to conclude the five-year survival rate.
2. We could not determine the causality of the related factors due to descriptive study.
3. Possibility of publication bias and overinterpretation due to only single case to be followed up and fortunately in this case showed good result.

In this case, the donor-recipient relationship is mother-daughter. The recipient was a 15-year-old girl with complaints of recurring low back pain since one year previously and had been diagnosed with hypertension and stage V chronic kidney disease with hemodialysis therapy two to three times a week for three months. Meanwhile, the donor was a 42-year-old recipient mother without a history of surgery or being hospitalized before. The HLA screening showed mismatch typing 3/6 and flow cytometry of T and B lymphocytes was negative. The renal transplantation was then carried out in March 2015.

It has been observed that patients with a greater risk of typing compatibility have a lower risk of acute rejection reactions as well as a better likelihood of transplant resistance. During this time, HLA typing was used with serology and then gradually adopted the molecular DNA method. ^17^ Most of the tissue typing laboratories started HLA typing with serology and then gradually adopted the molecular method, first for class II and then for class I. In this case, flow cytometry crossmatch and HLA typing based on DNA were used to predict graft rejection between the recipient and donor. ^18^

In a period of five years, this case showed good results with survival in a pediatric renal transplantation where the patient could continue normal activities and study at university. The patient's serum creatinine results ranged from 1.5-2 mg/dl and tacrolimus levels were also in the normal range between 3.5-7 ng/dl in these five years. Renal transplantation is thought to be more cost-effective than other renal replacement therapies such as lifelong hemodialysis, although direct economic analysis for renal transplantation in Indonesia has not been published.

A brief perspective from the patient about the procedure and the follow up would be a challenging part for this case. The patient told us how grateful she was to have a brand-new life with a new fresh kidney thus she could do anything which she could not do in a limited life before. She felt free to resume usual activity and the risk of graft rejection could be predicted and prevented by using the flow cytometry crossmatch and HLA-immunophenotyping based on DNA for screening test. Even though she had to undergo many parts of procedure, such as preoperative hemodialysis, kidney transplantation, redo surgery, DJ-stent removal, consumed many pills, came to urology clinic every month to get monitoring and evaluation of treatment and the upcoming follow ups, she never regretted being part of the procedure. She felt so grateful and thankful for the treatment she got from us. The best part of her perspective was this method brings the diseased patient into a new era of meaningful and independent life. She could do a jogging time, follow any meetings and activities in her university.

## Conclusion

The optimum follow-up results in the patient in this case suggest that renal transplantation in children can provide hope of survival of both the patient and the donor, even if this procedure is performed in developing countries, using the flow cytometry crossmatch screening method along with DNA-based HLA immunophenotyping.

## Data availability

All data underlying the results are available as part of the article and no additional source data are required.

## Consent

Written informed consent for publication of their details and clinical images was obtained from the patient.

## Ethics

Ethical approval for this case report was obtained from The Ethics Committee of Saiful Anwar General Hospital, Malang with approval number 400/006/CR/302/2020.
