# Supplementary material for: Case Report: A five-year follow up after pediatric renal transplantation using flow cytometry crossmatch and HLA immunophenotyping based on DNA for screening test
Source: F1000Res. 2021 May 6;10:352. [Version 1] doi: 10.12688/f1000research.51407.1 (PMC8493425; doi:10.12688/f1000research.51407.1)
Supplement: Peer review history

# Peer review: Reviewer response for version 1

**DOI:** 10.5256/f1000research.54570.r94677

**Published:** 2021-10-05

**Authors:** Shigeo Horie

**Affiliations:** 1 Department of Urology, Juntendo University, Tokyo, Japan

The authors reported the successful case of pediatric renal transplant after the first five-year follow-up. This was the first case using flow cytometry crossmatch and HLA immunophenotyping based on DNA for screening test in their institutions. They did a meticulous description of the case including surgical trouble, which will be quite useful for the readers. The background is well described. This case was well examined and the outcome of the transplant was excellent, reporting a good course of pediatric transplant.

 The methodological description of crossmatch was not well described. This is the only drawback of this paper. The authors should elaborate more on the detailed description of the method of flow cytometry crossmatch.

Are enough details provided of any physical examination and diagnostic tests, treatment given and outcomes?

Yes

Is the case presented with sufficient detail to be useful for other practitioners?

Yes

Is sufficient discussion included of the importance of the findings and their relevance to future understanding of disease processes, diagnosis or treatment?

Yes

Is the background of the case’s history and progression described in sufficient detail?

Yes

Reviewer Expertise:

Urology

I confirm that I have read this submission and believe that I have an appropriate level of expertise to confirm that it is of an acceptable scientific standard.

# Peer review: Reviewer response for version 1

**DOI:** 10.5256/f1000research.54570.r94891

**Published:** 2021-09-22

**Authors:** Nicolas Guillaume

**Affiliations:** 1 Department of Histocompatibility, Amiens University Medical Center, Amiens, France

The authors presented their first experience of renal transplantation using flow cytometry screening and HLA immunophenotyping. The authors cannot perform anti-HLA antibodies assays (screening and identification by CDC or Luminex assay). We have no information about the sensitivity of their flow cytometry crossmatch assay (the link between positivity and antibodies MFI by Luminex). They do not show at least a determination of antibodies screening by CDC. However, it is very unusual to publish this type of transplantation without antibodies data to perform at least a virtual crossmatch before flow cytometry crossmatch. In my opinion, this article cannot be indexed.

Are enough details provided of any physical examination and diagnostic tests, treatment given and outcomes?

No

Is the case presented with sufficient detail to be useful for other practitioners?

No

Is sufficient discussion included of the importance of the findings and their relevance to future understanding of disease processes, diagnosis or treatment?

No

Is the background of the case’s history and progression described in sufficient detail?

Yes

Reviewer Expertise:

Immunogenetics, transplantation (kidney, hematopoietic stem cells)

I confirm that I have read this submission and believe that I have an appropriate level of expertise to state that I do not consider it to be of an acceptable scientific standard, for reasons outlined above.

# Peer review: Reviewer response for version 1

**DOI:** 10.5256/f1000research.54570.r86508

**Published:** 2021-07-26

**Authors:** Nur Rasyid

**Affiliations:** 1 Department of Urology, Faculty of Medicine, Universitas Indonesia, Cipto Mangunkusumo Hospital, Jakarta, Indonesia

The authors presented their case report on a five-year follow-up after pediatric renal transplantation using flow cytometry crossmatch and HLA immunophenotyping based on DNA for screening test. This is a good case report, however some issues needed to be clarified further:

**Abstract:**

1. **Background: "The survival rate at one, five and ten years has been shown to be 99%, 97% and 96%...". **Please elaborate on what survival rate these numbers are for. The previous sentences were about flow cytometry combined with HLA immunophenotyping.
2. **Case presentation: "...and she could resume normal life". **Please use different vocabulary to define her quality of life post-transplantation.
3. **Conclusion: "Survival five years after the procedure showed a beneficial outcome of the method used." **Please elaborate further on the word "beneficial" to objective parameters that are evaluated during this five-year follow-up.

**Background:**

1. Please elaborate further on renal transplantation conditions in Indonesia. In general, the source of the donor in kidney transplants can be from cadaveric or living donors. In Europe and Western countries, cadaveric donors are more prevalent than living donors. This is interesting as in Indonesia there are no reports on cadaveric donors since the regulation of using cadaveric donors is still in progress. In this case report, the donor is from her mother (living donor). In my opinion, it would be nice if the authors mention this condition also in the Background. 
2. It would be better if you elaborate on why this is the first time your transplantation center used flow cytometry and HLA immunophenotyping. Is there a limitation of the healthcare facility or is it a procedure cost problem?
3. **Paragraph 4, Line 3-4: "...the prevalence of renal transplantation survival". **It would be better if you omit the words "the prevalence of".
4. Please include a sentence/paragraph explaining the optimal expected result for flow cytometry crossmatch combined with HLA immunophenotyping based on DNA

**Case presentation:**

1. **Paragraph 2, Line 1: **Please change the first sentence in your second paragraph to describe the condition when your team first met her, such as **"She came to the hospital with right and left lower back pain for the past year".**
2. **Paragraph 2, Line 6:** Please summarize the chest radiography expertise to only the important aspects. Normal values that are unrelated to renal transplantation can be omitted.
3. **Paragraph 2, Line 8:** Misspelling of " **nodul**", It is supposed to be "nodule".
4. **Paragraph 3. Line 1-3:** There was no need to re-mention laboratory values that support your diagnosis. You have previously mentioned those in Paragraph 2, except for GFR.
5. **Paragraph 4, Line 1:** You don't need to mention " **urologists from Malang...**", as it was already described in your author and affiliation list.
6. **Paragraph 4, Line 2:** **"The patient had been optimized by hemodialysis prior surgery..."** should be "...prior to surgery". Please change " **optimize**" to another word more suitable to refer to a patient. For example: "pre-treated". Furthermore, please further elaborate on the details of the pre-operative hemodialysis in your case.
7. **Paragraph 5, Line 3:** It is more appropriate not to use the drug brand name in this case report. Instead, you can use the drug's generic name.
8. **Paragraph 6, Line 2:"...by urologist from Malang"** is not necessary, as you have mentioned the author's affiliation beforehand.
9. **Paragraph 6, Line 3-4: "...creatinine level had increased by 4.9 g/dL"**. Please clarify whether the creatinine level rises to 4.9 g/dL or rises by 4.9 g/dL. Furthermore, the typical measurement unit for creatinine level is mg/dL. In your article, it was written in g/dL. Is this accurate or is it supposed to be mg/dL? Additionally, please use capital letters when typing "liter" in dL, instead of "dl" written in your article.
10. **Paragraph 6:** It would be great if you could provide us an endourology picture from the Holmium laser URS procedure.
11. **Paragraph 9, Line 2:** Please use the drug's generic name instead of its brand name.
12. **Paragraph 9, Line 6:** Previously, in Paragraph 2, you have used the term **"blood urea nitrogen (BUN)",** however, in this Paragraph you use the term "urea". It would be better to only use one term to maintain congruence.
13. While this paper focuses on the transplant recipient, it would be very enlightening if you could also provide information regarding the donor after five years of kidney donation. How is the donor's general wellbeing?

**Discussion:**

1. **Paragraph 1, Line 3**: Please revise **“longer survival”** to “better/longer life expectancy”.
2. **Paragraphs 2-3** could be further simplified or moved to the Background section to explain more about typing compatibility and its methods of examination.
3. **Paragraph 5, Line 3**: Please revise **“42-year-old recipient mother”** to “the donor was a 42-year-old female, who was the recipient’s mother…” to avoid misunderstanding.
4. **Paragraph 6, Line 1**: please revise **“greater risk” **to “greater/better match” to avoid misunderstanding.
5. **Paragraph 6**: It would be enlightening for the readers if ideal HLA typing compatibility and flow cytometry results for kidney transplantation from other studies are mentioned.
6. **Paragraph 7**: Please mention other studies that include yearly follow-up cases for kidney transplantation recipients to compare your result with other studies, such as QoL, laboratory results, and kidney function.

Are enough details provided of any physical examination and diagnostic tests, treatment given and outcomes?

Yes

Is the case presented with sufficient detail to be useful for other practitioners?

Yes

Is sufficient discussion included of the importance of the findings and their relevance to future understanding of disease processes, diagnosis or treatment?

Yes

Is the background of the case’s history and progression described in sufficient detail?

Partly

Reviewer Expertise:

Urology, Kidney Transplantation, Kidney Stone, Andro-Urology

I confirm that I have read this submission and believe that I have an appropriate level of expertise to confirm that it is of an acceptable scientific standard, however I have significant reservations, as outlined above.

# Peer review: Reviewer response for version 1

**DOI:** 10.5256/f1000research.54570.r84926

**Published:** 2021-06-09

**Authors:** Ali Ersin Zumrutbas

**Affiliations:** 1 Department of Urology, Pamukkale University School of Medicine, Denizli, Turkey

This case report was the first five-year follow-up of pediatric renal transplantation using flow cytometry crossmatch and HLA immunophenotyping based on DNA as a screening test in a local hospital. I would like to congratulate the authors for this case report including a comprehensive review and sufficient images. Minor grammatical and syntax errors should be corrected.

Are enough details provided of any physical examination and diagnostic tests, treatment given and outcomes?

Yes

Is the case presented with sufficient detail to be useful for other practitioners?

Yes

Is sufficient discussion included of the importance of the findings and their relevance to future understanding of disease processes, diagnosis or treatment?

Yes

Is the background of the case’s history and progression described in sufficient detail?

Yes

Reviewer Expertise:

Urology

I confirm that I have read this submission and believe that I have an appropriate level of expertise to confirm that it is of an acceptable scientific standard.
